# Hypercholesterolemia boosts joint destruction in chronic arthritis. An experimental model aggravated by foam macrophage infiltration

**DOI:** 10.1186/ar4261

**Published:** 2013-08-13

**Authors:** I Prieto-Potín, JA Roman-Blas, MJ Martínez-Calatrava, R Gómez, R Largo, Gabriel Herrero-Beaumont

**Affiliations:** 1 Bone and Joint Research Unit, Service of Rheumatology, IIS Fundación Jiménez Díaz, Universidad Autónoma, Av. Reyes Católicos 2, 28048, Madrid, Spain

## Abstract

**Objective:**

The aim of this study was to determine whether hypercholesterolemia increases articular damage in a rabbit model of chronic arthritis.

**Methods:**

Hypercholesterolemia was induced in 18 rabbits by administrating a high-fat diet (HFD). Fifteen rabbits were fed normal chow as controls. Chronic antigen-induced arthritis (AIA) was induced in half of the HFD and control rabbits, previously immunized, by intra-articular injections of ovalbumin. After sacrifice, lipid and systemic inflammation markers were analyzed in blood serum. Synovium was analyzed by Krenn score, multinucleated cell counting, immunohistochemistry of RAM11 and CD31, and TNF-α and macrophage chemoattractant protein-1 (MCP-1) gene expression. Active bone resorption was assessed by protein expression of receptor activator of nuclear factor kappa-B ligand (RANKL), osteoprotegerin (OPG) and quantification of cathepsin K, contact surface and the invasive area of pannus into bone.

**Results:**

Rabbits receiving the HFD showed higher total serum cholesterol, HDL, triglycerides and CRP levels than rabbits fed a normal diet. Synovitis score was increased in HFD, and particularly in AIA and AIA + HFD groups. AIA + HFD synovium was characterized by a massive infiltration of RAM11+ cells, higher presence of multinucleated foam cells and bigger vascularization than AIA. Cathepsin K+ osteoclasts and the contact surface of bone resorbing pannus were also increased in rabbits with AIA + HFD compared with AIA alone. Synovial TNF-α and MCP-1 gene expression was increased in AIA and HFD rabbits compared with healthy animals. RANKL protein expression in AIA and AIA + HFD groups was higher compared with either HFD or normal groups.

**Conclusions:**

This experimental model demonstrates that hypercholesterolemia increments joint tissue damage in chronic arthritis, with foam macrophages being key players in this process.

## Introduction

The increased burden of cardiovascular disease in rheumatoid arthritis (RA) is only explained partly by traditional cardiovascular risk factors. They seem to have similar prevalence in RA and non-RA patients, suggesting that other factors contribute significantly to this health issue [1,2]. In fact, non-traditional risk factors, such as RA disease activity/severity measures and some anti-rheumatic drugs, have been consistently associated with increased cardiovascular risk. Thus, RA inflammation plays a notable role in the development of cardiovascular disease.

Chronic synovitis is a source of inflammatory mediators that include cytokines, chemokines and adipokines, such as TNF-α, macrophage chemoattractant protein-1 (MCP-1), plasminogen activator inhibitor-1, interleukin (IL)-6 and others. In addition to the production of most of these pro-inflammatory cytokines, there is increasing evidence concerning the contribution of dysregulated adipose tissue through adipokine secretion to systemic RA inflammation [3]. Indeed, the inflammatory actions exerted by adipokines could explain some of the association between several rheumatic diseases and cardiovascular comorbidities [4]. A relevant role has been suggested for leptin in immunity, not only by maintaining energy homeostasis but also by regulating the function of immune cells. Specifically, leptin has been shown to promote phagocytic function and induce production of several pro-inflammatory cytokines in macrophages and monocytes [5]. Serum resistin levels have been shown to be higher in patients with RA than in healthy controls and correlate with inflammation and joint damage. In RA, macrophages, among other immune cells, showed co-localization with resistin [6]. An increase of leptin and resistin (pro-atherogenic hormones) and the decrease of adiponectin (anti-atherogenic hormone) may alter endothelial homeostasis in RA patients [7].

Endothelial dysfunction occurs in early stages of RA and atherosclerosis as a response to the raised expression of chemokines and adhesion molecules, promoting the enhancement of vessel wall permeability. These events favor leukocyte trafficking toward the site of synovial inflammation in arthritis [8] and/or promote infiltration of lipids, monocytes and T-lymphocytes. Further appearance of foam cells and fatty streaks within the vessel wall originates the formation of atherosclerotic plaques [9]. In addition, recent data showed that the vascular regenerative action of endothelial progenitor cells is altered in RA and atherosclerosis [10-13]. Therefore, the increase of pro-inflammatory signals and the impairment of reparatory processes, both mechanisms shared between RA and atherosclerosis, may contribute significantly to joint damage in RA.

The role of hypercholesterolemia in the progression of atherosclerosis is well established [14,15]. Severe hypercholesterolemia aggravated atherosclerotic plaques instability and aortic lesions mainly due to massive Mφ infiltration in our animal rabbit model of atherosclerosis associated with chronic arthritis [16]. Macrophages (Mφ) act as immune innate cells, antigen presenting cells and, finally, effector cells for joint inflammation in both acute and chronic phases. Indeed, Mφ contribute to the hyperplasia of the lining layer, and are the main cells in the mononuclear infiltration of the synovial sublining and the cartilage-pannus junction, as well as, the major producers of prominent inflammatory mediators. Activated Mφ may also differentiate into osteoclast-like cells and become involved in bone resorption [17-20]. Because relevant pathophysiological mechanisms occur similarly in both atherosclerosis and chronic inflammatory states, we hypothesize that hypercholesterolemia may also significantly contribute to joint damage in chronic arthritis through the enhancement of Mφ aggressiveness, as suggested in early findings in our previous study [16].

In this regard, significant research efforts have just begun to explore the mechanisms underlying the influence of hypercholesterolemia in the development and progression of inflammatory arthritis. During hyperlipidemia, adipocytes release well-known pro-inflammatory mediators and adipokines that play relevant roles in inflammatory arthritides [21]. A lipid-rich diet has been shown to contribute to the switch in polarization of adipose tissue Mφ from an anti-inflammatory (M2) to a pro-inflammatory (M1) state associated with obesity-induced insulin resistance [22]. This change induces systemic inflammation and potentially increases the progression of chronic arthritis. Hyperlipidemia has also been suggested to promote the osteoclastic potential of bone marrow cells *in vivo*. Indeed, the presence of lipid oxidation products in bone marrow and an increased osteoclast size in bones indicates a functional, but not numeric, difference in osteoclasts as described in hyperlipidemic mice [23]. Furthermore, diet-induced hypercholesterolemia in mice was associated with reduced bone quality measures that resemble human bone with osteoporosis [24]. Thus, hypercholesterolemia may start a systematic loss of bone homeostasis.

Therefore, in this study we determine whether hypercholesterolemia increases articular damage in a rabbit model of chronic arthritis and if this effect occurs through the activation of the synovial mononuclear phagocyte system.

## Material and methods

### Experimental model in rabbits

Adult male New Zealand rabbits with a body weight of 3 to 3.5 kg (Granja San Bernardo, Navarra, Spain) were used for the experimental procedures. Animal handling and experimentation were performed in accordance with Spanish Regulations and the Guidelines for the Care and Use of Laboratory Animals drawn up by the National Institutes of Health (Bethesda, MD, USA). The experimental protocol was approved by the Institutional Ethics Committee.

**Figure 1:**
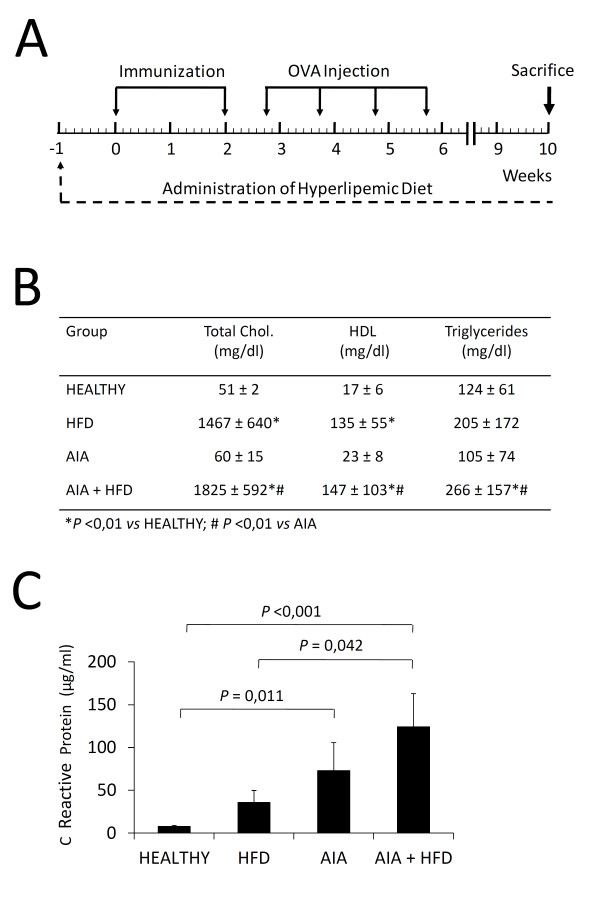
**Effect of the hyperlipemic diet and chronic antigen-induced arthritis (AIA) on serum content**. **A**, Schematic representation of the experimental model. **B**, Total cholesterol (Total Chol), high density lipoprotein (HDL) cholesterol and triglycerides levels (mg/dl) in the sera of healthy rabbits, high-fat diet (HFD) rabbits, AIA rabbits and AIA + HFD rabbits. **C**, Concentration of C reactive protein (µg/ml) in the sera of experimental rabbits. Bars show the mean and SEM (*n *= 7 to 9 rabbits per group). OVA, Ovalbumin.

After two weeks of adaptation to our facilities, 18 rabbits were fed *ad libitum *with a high-fat diet (HFD) consisting of 1% cholesterol + 3% peanut oil (Harlan, Inc., Indianapolis, IN, USA). One week later, antigen-induced arthritis (AIA) was induced in half of these animals (*n *= 9; AIA + HFD) according to a protocol previously described [16]. Briefly, animals were given two intradermal injections of 1 ml ovalbumin (OVA) (4 mg/ml; Sigma-Aldrich, St. Louis, MO, USA) in Freund's complete adjuvant (Difco, Detroit, MI, USA). Five days after the second injection, 1 ml of OVA (5 mg/ml in 0.9% NaCl) was injected intra-articularly into the knee joint on a weekly basis over the following four weeks (Figure 1A). The other half of HFD-fed rabbits underwent no injections (*n *= 9; HFD). We simultaneously induced AIA in a group of nine rabbits receiving standard chow. From this group, two rabbits died for unknown reasons and the remaining rabbits went through the whole study (*n *= 7; AIA). Eight additional rabbits fed with standard chow and spared from any experimental intervention were used as healthy controls.

At the end of the study, rabbits were bled from their marginal ear vein, and then euthanized with an overdose of pentobarbital (Braun Medical SA, Barcelona, Spain) in order to evaluate chronic damage. The synovial membranes of the right knee of each rabbit were fixed in 4% buffered paraformaldehyde and embedded in paraffin for histological studies to be performed. The synovial membranes from the left knees were immediately frozen and used for molecular biology studies. Both femurs were fixed, decalcified for four weeks in 10% formic acid plus 5% paraformaldehyde, and then embedded in paraffin. Articular cartilage and subchondral bone were obtained from both tibias for gene and protein expression studies.

### Serum chemistry

Ten milliliters of blood were used for serum extraction. Total and high-density lipoprotein (HDL) cholesterol and triglycerides were measured by Advia^®^2400 chemistry system (Siemens Healthcare Diagnostics, Tarrytown, NY, USA). A specific enzyme-linked immunosorbent assay kit was used to measure C-reactive protein (CRP; Alpha Diagnostic, San Antonio, TX, USA).

### Histopathology

Synovial histopathology was evaluated in hematoxylin-eosin (H-E) stained sections by two blinded observers, according to the Krenn scale, as previously described [25]. Briefly, lining hyperplasia, fibrovascular alterations at the interstitium, and the tissue infiltration were independently evaluated using 0 to 3-point subscales, where 0 indicates absence, 1 mild, 2 intermediate and 3 strong. The total score was obtained from the sum of partial grades with a maximum total score of 9 [26].

Multinucleated cell counting was performed in H-E sections by direct analysis of 10 randomly chosen microscopic fields, where lining and sublining layers were identifiable by the observer at 20x magnification.

In order to evaluate the invasiveness of the synovium at the posterior bone-pannus interface region, we quantified the extent of the contact surface between the pannus and bone/calcified cartilage, and the area of invasive pannus in contact with bone in H-E sections of femoral condyles. The microphotographs of the posterior bone-pannus interface region at 40x magnification of each rabbit were analysed. All samples were positioned in the same plane in order to assess a similar area in each measurement. For contact surface, the length of the boundary line between the pannus and bone/calcified cartilage tissue was measured. For the quantification of the invasive area, each tissue (bone, synovial membrane or cartilage) was identified by a distinctive color and then the area of each color was measured. The number of cathepsin K-positive osteoclasts was assessed in the same localization in order to estimate active bone resorption, and was expressed as positive cells per area. The assessment of these parameters was carried out with Image-Pro Plus software (version 4.5 for Windows, Media Cybernetics, Inc, Silver Spring, MD, USA).

### Immunohistochemistry

In the synovial membrane, we identified macrophages using a monoclonal anti-rabbit macrophage antibody (RAM11; Dako, Glostrup, Denmark), according to protocol [27]. The antibody was detected with a biotinylated goat anti-mouse IgG (1:200; Amersham, Arlington Heights, IL, USA) visualized with a horseradish peroxidase/ABComplex using 3,3diaminobenzidine tetra-hydrochloride as the chromogen (Dako, Camarillo, CA, USA). The tissues were counterstained with hematoxylin and mounted in DPX medium (VWR International, Leuven, Belgium). Computer-assisted analysis was performed with Leica DMD108 Digital Micro Imaging device (Leica, Microsystems, Inc., Buffalo Grove, IL, USA) and with Image-Pro Plus software (version 4.5 for Windows, Media Cybernetics, Inc, Silver Spring, MD, USA)). The results were expressed as percentage of positive area. An IgG isotype was used as a negative control.

CD31 immunostaining (Abcam, Cambridge, UK) was assessed in the synovial membrane, as a marker of vascular endothelial cells. Briefly, synovium sample slides were scanned in the Coreo Iscan Au scanner (Ventana Medical Systems, Tucson, AZ, USA) and then total cells, CD31-positive cells and the entire sample area were assessed with Virtuoso Image management software (Ventana Medical Systems, Tucson, AZ, USA). The results were expressed as the ratio between CD31positive cells and the area in mm^2^.

Cathepsin K-positive cells were evaluated in femur sections in order to assess bone resorption (Abcam, Cambridge, UK). Briefly, the number of cathepsin K-positive multinucleated cells was assessed at the bone-pannus interface region of AIA and AIA + HFD knees. All samples were positioned in the same plane in order to assess a similar area in each measurement, and the results are shown as positive staining per area.

### Gene expression analysis

Total RNA was isolated from synovial membranes using TriPure Isolation Reagent (Roche Diagnostics, Indianapolis, IN, USA), according to the manufacturer´s instructions. A total of 1 µg RNA was reverse-transcribed with the high capacity cDNA kit (Applied Biosystems, Foster City, CA, USA) and RNA expression was quantified by single-reporter real time PCR using the StepOnePlus™ detection system and StepOne™ software v2.2 (Applied Biosystems). Specific oligonucleotide fluorescently labeled primers, TaqMan FAM probe assay for TNF-α (Oc 03397715_m1) and assay-on-demand for MCP-1 were purchased from Applied Biosystems. A pre-designed TaqMan FAM probe assay for GAPDH (Oc 03823402_g1, Applied Biosystems) was also used as an endogenous control, and mRNA expression was normalized to GAPDH RNA in each well.

### Western blot analysis

Tissues were homogenized in liquid nitrogen, and total proteins were extracted employing an extraction buffer containing 15 mM HEPES, 10% glycerol, 0.5% NP-40, 250 mM NaCl, 1 mM EDTA, 1:1,000 phenylmethanesulfonylfluoride (PMSF) and a protease inhibitor cocktail (Sigma-Aldrich). Protein concentration was determined as previously described [28], and subsequently, 20 μg of total protein from each tissue was resolved on 15% acrylamide-SDS gels. After transfer to polyvinylidene difluoride (PVDF) membranes (Millipore, Molsheim, France) in 48 mM Tris, 39 mM glycine and 20% methanol buffer at 20 V for 1 h at room temperature, membranes were blocked in 5% skimmed milk in PBS-Tween 20 for 1 h at room temperature and incubated overnight at 4°C with anti-receptor activator of nuclear factor kappa-B ligand (RANKL) antibodies (Peprotech, Neuilly-Sur-Seine, France) and anti-osteoprotegerin (OPG) (R&D Systems, Minneapolis, MN, USA) at 1/1,000 dilution each. Antibody binding was detected by enhanced chemoluminescence using peroxidase-labeled secondary antibodies, and the results were expressed as arbitrary densitometric units (AU). Loading control was performed on 15% acrylamide-SDS gels by employing EZBlue gel staining reagent (Sigma-Aldrich).

### Statistical analysis

All statistical analyses were performed using SPSS version 17.0 software for Windows (SPSS, Chicago, IL, USA), and results were expressed as the mean ± standard error of mean (SEM). The data from multiple groups were compared using a Kruskal-Wallis nonparametric test, and a pairwise comparison using the Mann-Whitney test was applied when overall differences were identified. *P-*values <0.05 were considered significant.

## Results

### Metabolic profile

Rabbits fed with a hyperlipemic diet (the HFD and AIA + HFD groups) showed significant higher levels of total serum cholesterol, HDL and triglycerides than healthy rabbits (*P *= 0.01). Likewise, the AIA + HFD group had higher lipid levels compared with the AIA group (Figure 1B). A decrease of rabbit weight (kg) in the HFD, AIA and AIA + HFD groups (3.4 ± 0.19; 3.23 ± 0.22 and 3.28 ± 0.12) was observed when compared to healthy rabbits (4.28 ± 0.06; *P *<0.002), at the time of sacrifice.

### CRP levels

Levels of CRP were significantly increased in sera of AIA and AIA + HFD groups (73.07 ± 32.58 and 124.2 ± 38.69) in comparison with the healthy group (7.83 ± 1.28, *P *= 0.011 and *P *<0.001, respectively). In addition, CRP levels of the AIA + HFD group were higher than the HFD group (124.2 ± 38.69 versus 35.71 ± 13.98, *P *= 0.042). No significant difference was found between the AIA and AIA + HFD groups; however, an increased tendency is observed in the AIA + HFD group (Figure 1C).

### Increase of synovial inflammation

**Figure 2:**
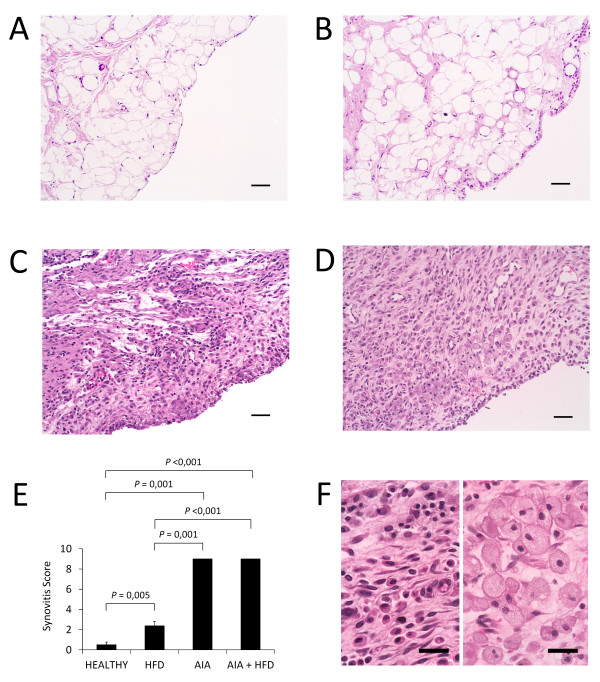
**Histopathology of the synovial membrane**. **A-D**, Representative sections of synovial membranes stained with hematoxylin and eosin from healthy rabbits (A), high fat diet (HFD) rabbits (B), chronic antigen-induced arthritis (AIA) rabbits (C), and HFD+AIA rabbits (D), original magnifications x200, scale bar = 50 µm. **E**, Global synovitis score, quantified as described in materials and methods. Bars show the mean and SEM (*n *= 7 to 9 rabbits per group). **F**, Detail of the inflammatory cells observed in the AIA synovium (left side) and in the AIA + HFD synovium (right side), original magnifications x630, scale bar = 25 µm. Inflammatory foam cells of AIA + HFD synovial membrane show an increased size compared with inflammatory cells of AIA synovium.

Histological changes of the synovial membrane were evaluated by the Krenn synovitis score. Synovial lesions were observed in HFD group compared with healthy rabbits (*P *= 0.005). AIA and AIA + HFD rabbits showed much more severe synovial changes than control groups, including lining hyperplasia, mononuclear cell infiltration at the sublining layer and lymphoid aggregates, occasionally organized into nodule-like structures (Figure 2). These synovial features mirror those present in rheumatoid synovium [29].

### Massive Mφ infiltration and presence of foam cells

**Figure 3:**
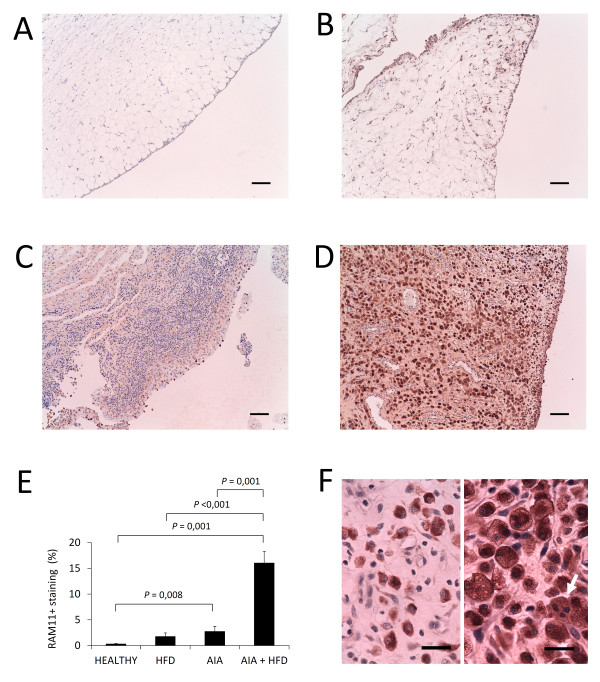
**Immunostaining of synovial macrophages**. **A-D**, Representative sections of synovial membranes stained with a monoclonal anti-rabbit macrophage antibody (RAM11) from healthy rabbits (A), high fat diet (HFD) rabbits (B), chronic antigen-induced arthritis (AIA) rabbits (C) and HFD + AIA rabbits (D), original magnifications x100, scale bar = 100 µm. **E**, Densitometric analysis of RAM11 staining percentage in the synovium of each group of animals. Bars show the mean and SEM (*n *= 7 to 9 rabbits per group). **F**, Detail of the RAM11-positive cells observed in AIA synovium (left side) and in the HFD + AIA synovial membrane (right side), original magnifications x630, scale bar = 25 µm. RAM11-positive foam cells of increased size, some of them with three nuclei (white arrow), are present in the HFD + AIA synovium.

Although both AIA and AIA + HFD rabbits presented the highest synovitis score, AIA + HFD synovium was characterized by a massive infiltration of RAM11-positive cells whose presence was greater than AIA and control synovial membranes (*P *= 0.001; Figure 3E). This remarkable feature along with the notable and consistent presence of foam cells in AIA + HFD synovium (Figures 2F and 3F), detected by Oil Red-O staining (data not shown), demonstrates a clear qualitative difference between synovial membranes of similar inflammation score grade from the AIA + HFD and AIA groups. The presence of foam cells was detected in the sublining and lining layers; the latter may be considered as a pathognomonic feature of the AIA + HFD group.

### Increase of TNF-α and MCP-1 gene expression

The HFD, AIA and AIA + HFD groups showed a three-fold increase of TNF-α gene expression levels (3.57 ± 0.73; 3.17 ± 1.25 and 2.44 ± 0.26) when compared with healthy rabbits (1.16 ± 0.24; *P *= 0.005; *P *= 0.05 and *P *= 0.005). MCP-1 gene expression levels of HFD and AIA groups were also increased when compared to the healthy group (2.59 ± 0.54; 3.50 ± 1.42 vs. 1.04 ± 0.17; *P *= 0.006; *P *= 0.016). AIA + HFD MCP-1 gene expression levels were lower than AIA (1.54 ± 0.22 vs. 3.50 ± 1.42; *P *= 0.048) (Additional file 1).

### Presence of multinucleated cells resorbing adipose tissue

**Figure 4:**
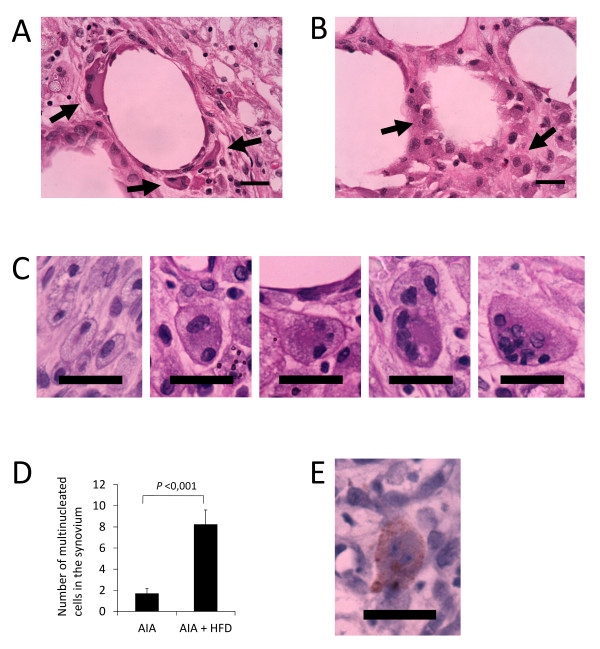
**Multinucleated cells resorbing fat tissue**. **A**, Multinucleated cells in crown-like structure surrounding adipocytes in the synovium of high fat diet (HFD) and chronic antigen induced arthritis (AIA) rabbits (black arrows, hematoxylin and eosin staining, original magnifications x630, scale bar = 25 µm). **B**, Multinucleated foam cells resorbing adipocytes structures in the synovium of HFD + AIA rabbits (black arrows, hematoxylin and eosin staining, original magnifications x630, scale bar = 25 µm). **C**, Progression of multinucleated cells presenting from two up to multiple nuclei in the synovium of HFD + AIA rabbits. **D**, Quantification of multinucleated cells in the synovial membranes of AIA and HFD + AIA rabbits. Bars show the mean and SEM (*n *= 7 to 9 rabbits per group). **E**, Circulating synovial cathepsin K-positive multinucleated cells in the proximity of pannus region (original magnification x630, scale bar = 25 µm).

Macrophages arranged in crown-like structures around adipocytes were observed in HFD, AIA and AIA + HFD synovium (Figure 4A, B). Interestingly, we found more frequently multinucleated cells engulfing adipocyte structures in the AIA + HFD group (Figure 4B), and even more progression of multinucleated cells, going from two up to multiple nuclei (Figure 4C). The number of multinucleated cells was significantly increased in AIA + HFD synovium (*P *<0.001; Figure 4D). Circulating synovial multinucleated cells were also observed in the proximity of the region, some of them were cathepsin K-positive (Figure 4E).

### Increase of angiogenesis

**Figure 5:**
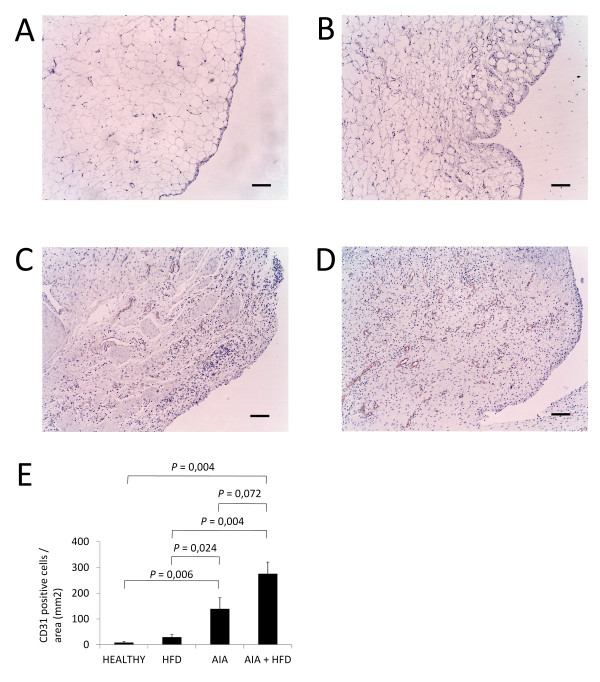
**Angiogenesis immunostaining in the synovial membrane**. **A-D**, Representative sections of synovial membranes stained with a monoclonal to CD31 antibody from healthy rabbits (A), high fat diet (HFD) rabbits (B), chronic antigen induced arthritis (AIA) rabbits (C) and HFD + AIA rabbits (D), original magnifications x100, scale bar = 100 µm. **E**, Semi-quantification of CD31-positive vascularization in the synovium of each group of animals. Bars show the mean and SEM (*n *= 7 to 9 rabbits per group).

Immunohistochemical analysis of synovial membranes for CD31 confirmed an increased vascularization by a higher number of vessels stained in AIA + HFD synovium compared with AIA synovium. Both groups showed by far more CD31-positive vessels than the HFD and healthy groups (Figure 5A, E). In addition, no difference was observed for CD31 staining between healthy and HFD groups; however, a tendency of more CD31 staining can be observed in HFD synovium.

### Increase of active bone resorption

**Figure 6:**
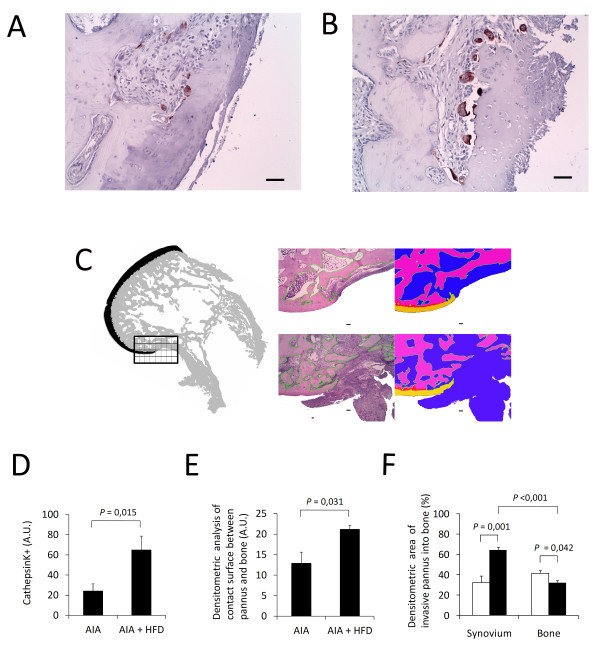
**Bone active resorption**. **A **and **B**, Representative sections of the bone-pannus interface region with cathepsin K-positive cells shows bone resorbing activity in chronic antigen-induced arthritis (AIA) rabbits (A) and HFD + AIA rabbits (B), original magnification x200, scale bar = 50 µm. **C**, Illustration of the region of interest evaluated in each femoral condyle at the posterior bone-pannus interface region. Contact surface and invasive areas were analysed in hematoxylin and eosin (H-E) stained sections and then photographed in chronic antigen-induced arthritis (AIA) rabbits (upper sections) and HFD + AIA rabbits (lower sections) (left side: H-E stained sections, right side: photographed sections, original magnification x400, scale bar = 100 µm). **D**, Densitometric analysis of cathepsin K-positive staining in the synovium of both groups of animals. Bars show the mean and SEM (*n *= 7 to 9 rabbits per group). **E **and **F**, Densitometric analysis of contact surface and invasive areas of AIA and HFD + AIA rabbits. White bars correspond to AIA group and black bars to AIA + HFD group; both show the mean and SEM (*n *= 7 to 9 rabbits per group).

We estimated active bone resorption by quantifying cathepsin K-positive osteoclasts and by determining the contact surface between pannus and bone/calcified cartilage and the area of invasive pannus into bone. Our results showed an increased presence of cathepsin K-positive osteoclasts in the synovium of AIA + HFD rabbits compared with that of AIA rabbits (*P *= 0.015; Figure 6A, B). We also observed a higher surface of bone resorbing pannus in AIA + HFD than in AIA rabbits (Figure 6C, F). We selected the posterior pannus-bone area as the region of interest to be measured because osteoclast differentiation from macrophages and activation within the aggressive pannus and subsequent subchondral bone invasion occur in this interface.

### Increase of RANKL protein levels

Both RANKL and OPG are expressed in cartilage, bone and synovium, as previously described [30]. RANKL expression in the HFD group was increased in all tissues when compared to the healthy group (2.49 ± 0.29 vs. 0.99 ± 0.18, *P *= 0.001 in cartilage; 4.10 ± 0.46 vs. 2.26 ± 0.34, *P *= 0.021 in bone; and 7.06 ± 1.01 vs. 3.04 ± 0.34, *P *= 0.015 in synovium). RANKL expression levels in both AIA and AIA + HFD groups were highly increased in all tissues when compared with the controls, healthy and HFD groups. Specifically in the synovium, AIA and AIA + HFD groups showed similar levels of RANKL protein expression (24.79 ± 2.23 and 24.63 ± 2.05) and were significantly higher compared with their controls, healthy and HFD groups (3.04 ± 0.34, *P *<0.001 and 7.06 ± 1.01, *P *<0.001, respectively) (Additional file 2).

## Discussion

This is the first *in vivo *study that demonstrates the harmful effect of hypercholesterolemia on the severity of chronic arthritis. Indeed, we characterized a novel model of chronic arthritis aggravated by hypercholesterolemia, which is driven by foam Mφ with an aggressive phenotype. These Mφ markedly damaged synovial membrane, calcified cartilage, juxta-articular bone and fatty tissue, and increased angiogenesis was also evidenced in the synovial membrane of our experimental animals. Likewise, reduced total cholesterol and HDL levels, as well as increased low-density lipoprotein (LDL)/vLDL, IL-6, sRANKL and CCL5 circulating levels mirroring RA patients was observed in the *K/BxAg7 *mice, a novel animal model of erosive arthritis followed by prominent aortic atherosclerosis. Remarkably, etanercept administration reduced arthritis and atherosclerosis development in these mice [31].

We found a modest, although evident, increase of synovial cell infiltration in non-arthritic rabbits fed with hypercholesterolemic diet. Accordingly, an atherogenic lipid profile characterized by high total cholesterol, triglyceride and apo B levels, and low HDLc levels was more prevalent in blood donors who later developed RA [32]. Similarly, a higher risk of moderate to severe RA was found in patients with metabolic syndrome [33]. Recent obesity studies have supported these findings since the lipid profile may also be influenced by weight/body mass index. Obesity has been associated with a modest risk for developing RA, but the rapid increase in obesity prevalence may be responsible for much of the increase in RA incidence [34]. Moreover, obesity was associated with worse RA disease outcomes, high prevalence of comorbidities and the poor therapy effect of glucocorticoids, disease-modifying antirheumatic drugs (DMARDs) and biologic agents [35-37]. All these findings suggest that hyperlipidemia may influence the initiation and progression of chronic inflammatory synovitis as well as indicate the potential relevance of its pharmacology modification.

Since there are no clearly designed studies showing that hypercholesterolemia increases chronic synovitis, investigating the effects of hypolipidemic statins on the course of RA may provide additional clues to this relationship. Besides their well-established beneficial effects on lipid metabolism in patients with hypercholesterolemia, cardiovascular diseases and inflammatory diseases [38-40], statins have also been shown to exert anti-inflammatory, antioxidant, immunomodulatory and antithrombotic effects [41]. A six-month clinical trial reported a modest but significant clinical improvement in RA subjects treated with atorvastatin [39]. Interestingly, a recent large population-based cohort study has shown an association between good adherence to statin treatment and reduced risk of developing RA [42]. Likewise, statins reduced the risk for the development of RA between 30% and 40%, although lipid lowering drugs other than statins were not associated with a decreased risk [43]. Hence, statins may be protective against development and progression of RA in those with hyperlipidemia.

Massive infiltrates of RAM11-positive macrophages, a major number of multinucleated cells and the presence of foam-like cells were characteristically seen in the AIA + HFD synovium. Activation of blood mononuclear cells as shown by enhanced nuclear factor-kappa B (NF-κB) transcription binding and massive Mφ infiltration in atherosclerotic plaques was previously described in the combined model [16]. Taken together, these findings suggest that the activation of peripheral monocytes could lead to systemic inflammation producing a huge Mφ tissue infiltration, accounting for the synovitis aggressiveness observed in this experimental model. In accordance, several quantitative immunohistological studies have highlighted the relevance of Mφ in driving the inflammatory response. The number of Mφ was increased in clinically affected joints compared with non-affected joints [44], and correlated well with clinical signs of disease activity, including joint pain and swelling [18]. Of relevance, the change in the number of sublining Mφ has been proposed as a sensitive biomarker to predict possible efficacy of new anti-rheumatic treatment [45].

The high plasticity of macrophages, as shown by the marked transformation of macrophages into foam cells, lipid-engulfed multinucleated cells and further cathepsin K-stained osteoclasts, was one of the major findings in hyperlipidemic rabbits with chronic arthritis. As described in the adipose tissue of obese mice and humans [17,46], macrophages were frequently observed around dead adipocytes forming characteristic 'crown-like' structures. This shows an exacerbated phagocytosis of dead adipocytes by activated macrophages in the sublining of AIA + HFD rabbits. The increased transformation of activated macrophages into foam cells in the lining and sublining layers of hyperlipemic animals with AIA occurs as a result of the enhanced uptake by their scavenger receptors of oxidized low density lipoproteins (oxLDL). A disruption of the transcriptional equilibrium mediated by lipid sensors between lipid metabolism and immune functions in macrophages promotes the enhancement of pro-inflammatory signals during their activation, and finally leads to formation of foam cells [17]. A persistent imbalance between pro- and anti-inflammatory mediators released by macrophages and/or deficient clearance of apoptotic foam cells can perpetuate chronic inflammation [47]. Indeed, macrophages from experimental animals generated significant levels of TNF-α gene expression and, potentially, other inflammatory mediators, such as IL-6 signaling, that may have induced C-reactive protein expression [16].

The growth of the synovial membrane was accompanied by neovascularization, as demonstrated by the increased CD31+ staining of blood vessels in AIA and, particularly, in AIA + HFD synovial membranes. Activated macrophages promote angiogenesis by the production of angiogenic factors, including pro-inflammatory cytokines, growth factors and chemokines that promote endothelial cell adhesion, receptor expression and neovascularization [48]. Many of these molecules were not induced in adipose tissue macrophages from C-C chemokine receptor 2 (CCR2) knockout mice fed with a high-fat diet, supporting the importance of CCR2 in regulating recruitment of inflammatory activated macrophages during obesity [49]. Successful treatment of RA with anti-TNF antibodies reduced levels of pro-angiogenic factors, including vascular endothelial growth factor (VEGF), and led to normalization of the vasculature. In addition, leptin has been suggested to induce angiogenesis in endochondral ossification [50]. These data emphasize the close links among angiogenesis, inflammation and obesity in this disease. In our animal model, whether hypercholesterolemia boosts angiogenesis in the synovium of arthritic rabbits remains to be established and ongoing research is being carried out in this regard.

The phenotype transformation of macrophages to cathepsin K-stained osteoclasts observed in AIA and, particularly, in AIA rabbits fed with a hyperlipemic diet remarkably resembles the development of osteoclasts from monocytes/macrophage precursor cells, one of the unique characteristic of RA [7]. Cathepsin K is the major protease of osteoclasts responsible for bone resorption and its specific degradation product, C-terminal telopeptide of collagen type I (CTX-I), has been extensively used as a surrogate measure of bone resorption [7,10,51-53]. Thus, the high active bone resorption found in AIA + HFD synovial membranes shows the influence of hyperlipidemia on bone metabolism. In fact, human macrophage foam cells produce CTX-I fragments in atherosclerotic plaques [19,20,22,23,53,54]. Multiple parameters of bone quality were altered by a hypercholesterolemic diet in mice, including bone mineral density (BMD) bone volume fraction, number of trabeculae and trabecular spacing, along with changes in the mechanical properties of the bone and an increase in bone resident osteoclasts [24]. Although synovitis score and the expression levels of RANKL, OPG and TNF-α were similar in AIA and AIA + HFD groups, bone resorption activity was remarkably increased in AIA + HFD rabbits suggesting a greater RANKL activity or efficiency in the presence of high LDL levels. It has recently been described that LDL deficiency causes impaired osteoclastogenesis with a subsequent decrease in bone resorption parameters and increased bone mass in mice due to a defect in osteoclastic cell-cell fusion [55]. In addition, Wnt-mediated signals might be altered in hyperlipidemia and, subsequently, affect bone resorption [56]. Thus, hypercholesterolemia may directly disrupt bone homeostasis.

## Conclusions

These findings provide a valuable piece of information about the hypercholesterolemia pathogenicity at the synovial membrane and cartilage-pannus junction. The hyperlipidemia-boosted systemic inflammation markedly damages articular tissues by macrophage massive infiltration and transformation to foam cells and active osteoclasts. In this context, Mφ accumulation at the site of inflammation may be a direct response to the abnormal fat metabolism caused by the increasing adiposity. Therefore, this experimental model shows that hypercholesterolemia markedly increments joint tissue damage in chronic arthritis, demonstrating a key role of synovial macrophages in this process, as well as their potential as a suitable target for successful RA therapy.

## Abbreviations

AIA: antigen induced arthritis; apoB: apolipoprotein B; BMD: bone mineral density; CCL5: C-C chemokine ligand 5; CCR2: C-C chemokine receptor 2; CRP: C reactive protein; CTX1: C terminal telopeptide of collagen type I; DMARDs: disease-modifying anti-rheumatic drugs; EDTA: ethylenediaminetetraacetic acid; GAPDH: glyceraldehydes 3-phosphate dehydrogenase; HDL: high density lipoprotein; H-E: hematoxylin and eosin; HEPES: hydroxyethylpiperazine-1-ethanesulfonic acid; HFD: high fat diet; IgG: immunoglobulin G; IL6: interleukin 6; LDL: low density lipoprotein; M1: pro-inflammatory macrophage; M2: anti-inflammatory macrophage; MCP-1: monocyte chemoattractant protein 1; MΦ: macrophage; NFkB: nuclear factor kappa B; NP: nonyl phenoxypolyethoxylethanol; OPG: osteoprotegerin; OVA: ovalbumin; oxLDL: oxidized low density lipoprein; PBS: phosphate buffered saline; PMSF: phenylmethanesulfonylfluoride; PVDF: polyvinylidene difluoride; RA: rheumatoid arthritis; RAM11: anti-rabbit macrophage monoclonal antibody; RANKL: receptor activator nuclear kappa B ligand; SDS: sodium dodecyl sulfate; SEM: standard error mean; sRANKL: soluble receptor activator nuclear kappa B ligand; TNF-α: tumor necrosis factor α; VEGF: vascular endothelial growth factor; vLDL: very low density lipoprotein

## Competing interests

The authors declare that they have no competing interests.

## Authors' contributions

GH-B, JAR-B, IP-P and RL were involved in the study conception and design. IP-P, MJM-C and RG were involved in the acquisition of data. IP-P, JAR-B, MJM-C, RG, RL and GH-B were involved in the analysis and interpretation of data. GH-B had full access to all study data and takes responsibility for the integrity and accuracy of data analysis. All authors were involved in drafting the article or revising it critically for important intellectual content. All authors approved the final version to be published.

## Supplementary Material

Additional file 1 **High fat diet (HFD) and antigen induced arthritis (AIA) interventions increase TNF-α (A) and MCP-1 (B) gene expression in the synovium**. Gene expression was examined by real time PCR and results are expressed as fold induction. Bars show the mean and SEM (*n *= 7 to 9 rabbits per group). Click here for file

Additional file 2 **RANKL and OPG protein expression**. **A, C **and **E**, Densitometric analysis of receptor activator nuclear kappa B ligand (RANKL) and osteoprotegerin (OPG) protein expression in cartilage (A), subchondral bone (C) and synovium (E). Bars show the mean and SEM (*n *= 7 to 9 rabbits per group). **B, D **and **F**, Representative Western blot images for RANKL and OPG detection in cartilage (B), subchondral bone (C) and synovium (F). EZ Blue-stained gels used as protein loading controls are also shown for healthy rabbits, high fat diet (HFD) rabbits, chronic antigen induced arthritis (AIA) rabbits and HFD + AIA rabbits. Click here for file
