# Supplementary figures and images for: Hypercholesterolemia boosts joint destruction in chronic arthritis. An experimental model aggravated by foam macrophage infiltration
Source: Arthritis Res Ther. 2013 Aug 13;15(4):R81. doi: 10.1186/ar4261 (PMC3978700; doi:10.1186/ar4261)

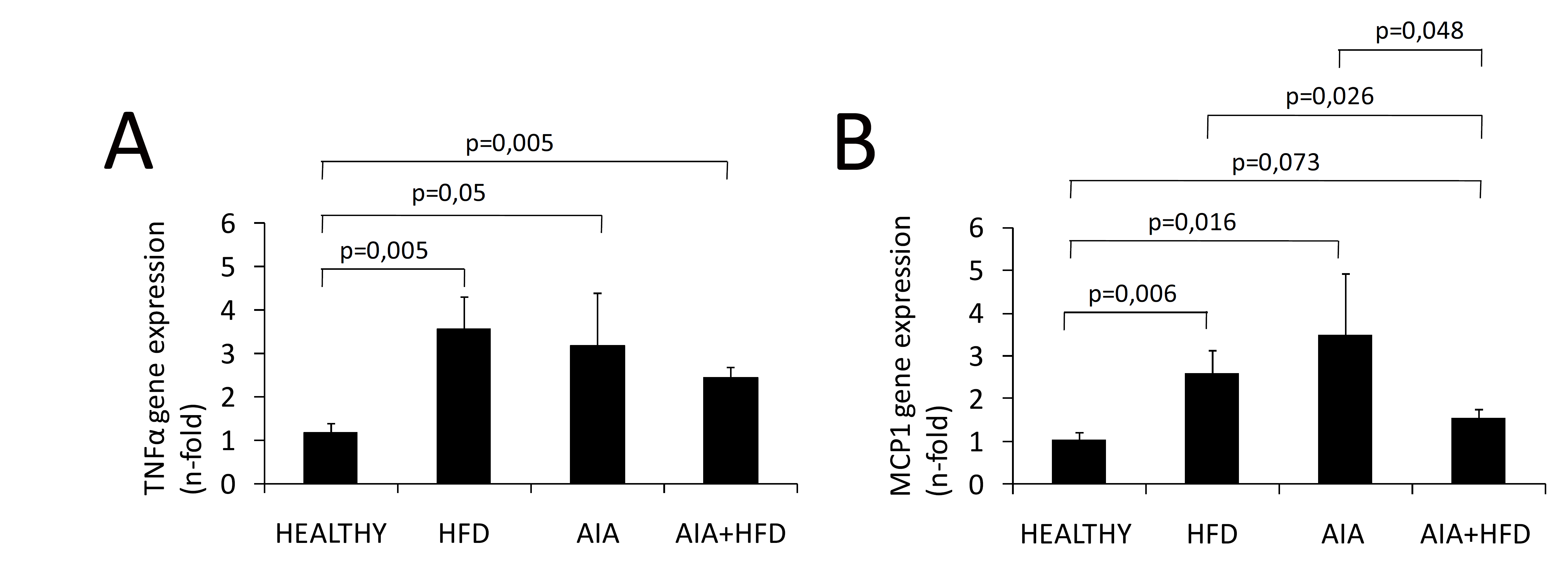

Supplement: Additional file 1 — High fat diet (HFD) and antigen induced arthritis (AIA) interventions increase TNF-α (A) and MCP-1 (B) gene expression in the synovium. Gene expression was examined by real time PCR and results are expressed as fold induction. Bars show the mean and SEM (n = 7 to 9 rabbits per group). [file ar4261-S1.TIFF]

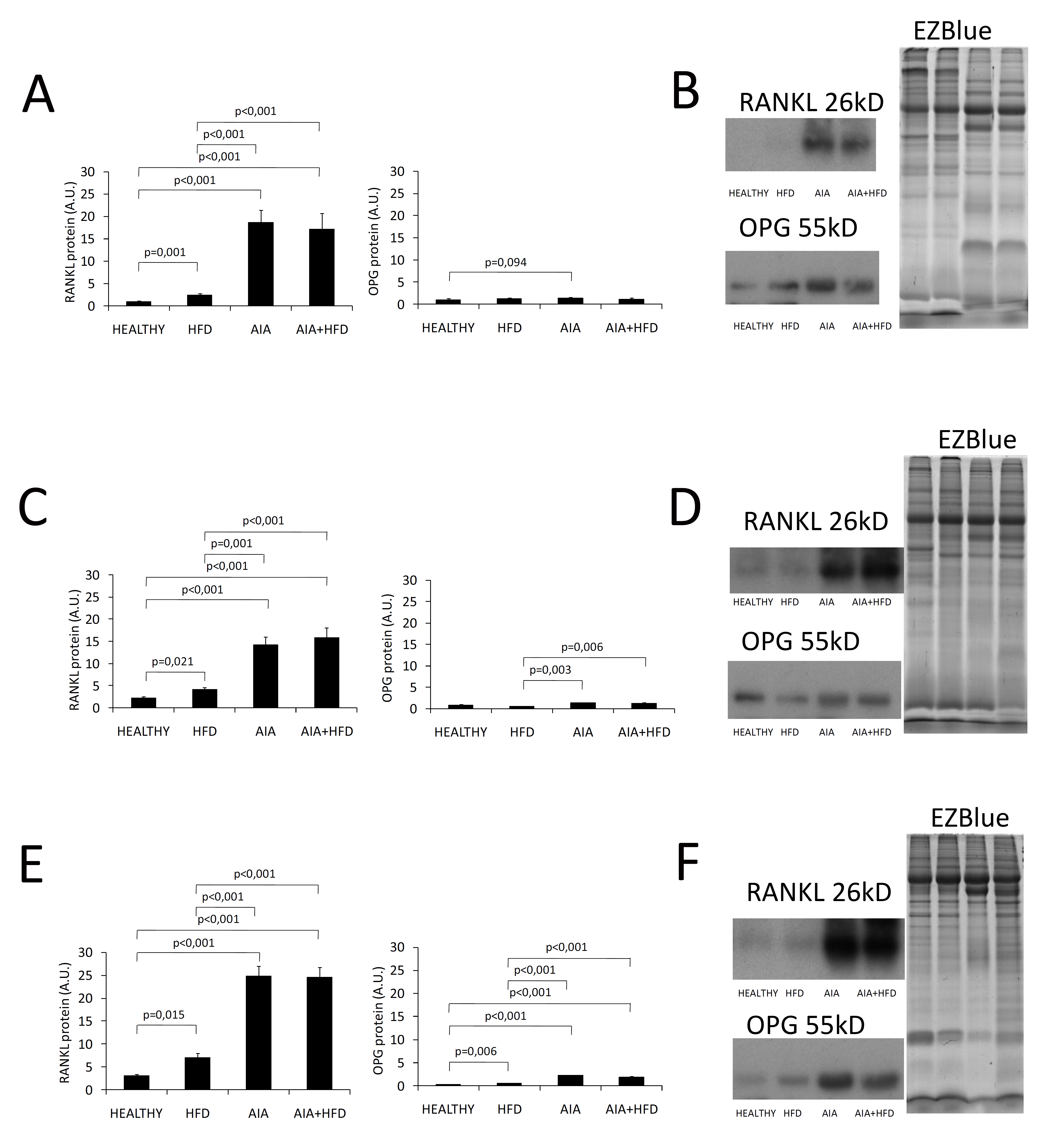

Supplement: Additional file 2 — RANKL and OPG protein expression. A, C and E, Densitometric analysis of receptor activator nuclear kappa B ligand (RANKL) and osteoprotegerin (OPG) protein expression in cartilage (A), subchondral bone (C) and synovium (E). Bars show the mean and SEM (n = 7 to 9 rabbits per group). B, D and F, Representative Western blot images for RANKL and OPG detection in cartilage (B), subchondral bone (C) and synovium (F). EZ Blue-stained gels used as protein loading controls are also shown for healthy rabbits, high fat diet (HFD) rabbits, chronic antigen induced arthritis (AIA) rabbits and HFD + AIA rabbits. [file ar4261-S2.TIFF]
